# Statin Use Is Associated With Disseminated Superficial Actinic Porokeratosis: A Population-Based Analysis

**DOI:** 10.7759/cureus.111144

**Published:** 2026-06-19

**Authors:** Dora R Goldstein, Kritin K Verma, Orson T Robertson, Kevin T Nguyen, Sahil Kapur, Daniel P Friedmann, Michelle B Tarbox

**Affiliations:** 1 School of Medicine, Texas Tech University Health Sciences Center El Paso Paul L. Foster School of Medicine, El Paso, USA; 2 School of Medicine, Texas Tech University Health Sciences Center, Lubbock, USA; 3 Dermatology, University of Toledo College of Medicine and Life Sciences, Toledo, USA; 4 Westlake Dermatology Clinical Research Center, Westlake Dermatology & Cosmetic Surgery, Austin, USA; 5 Dermatology, Texas Tech University Health Sciences Center, Lubbock, USA

**Keywords:** dermatologic side effects, disseminated superficial actinic porokeratosis (dsap), lipid-lowering therapy, trinetx database, statins

## Abstract

Statins are lipid-lowering drugs that block 3-hydroxy-3-methylglutaryl-coenzyme A reductase to prevent atherosclerotic cardiovascular disease. While statins are generally well tolerated, they have been linked to a variety of dermatological disorders. This study investigated the relationship between statin use and disseminated superficial actinic porokeratosis (DSAP), a chronic hereditary skin condition marked by hyperkeratotic papules with characteristic ridge-like borders. Using the TriNetX database, we conducted a retrospective cohort study of 970,166 statin medication patients identified by RxNorm codes. DSAP cases were identified using diagnostic codes, and propensity score matching was used to balance demographic variables between the treatment and control groups. Among statin-treated patients, 660 (0.068%) cases of DSAP were found, compared with 478 (0.049%) cases in controls. Statin medication was linked to a significant increase in DSAP risk (RR, 1.381; 95% CI, 1.227-1.553; p=0.0001). This study finds a statistically significant correlation between statin use and DSAP, emphasizing the significance of monitoring for dermatologic side effects in statin-treated patients.

## Introduction

Statins, lipid-lowering medications that inhibit 3-hydroxy-3-methylglutaryl-coenzyme A reductase, are widely used to reduce low-density lipoprotein cholesterol levels and decrease the risk of atherosclerotic cardiovascular disease [1]. While statins are generally well-tolerated, they have been implicated in various dermatologic conditions [2]. One condition is disseminated superficial actinic porokeratosis (DSAP), a chronic, rare, inherited skin disorder affecting approximately 1%-4% of the general population, characterized by multiple small, annular, hyperkeratotic papules with a distinctive ridge-like border [3,4]. DSAP, a subtype of porokeratosis characterized by abnormal keratinization, has been linked in prior literature to possible metabolic dysregulation, though its exact etiology remains unclear [4]. Given that statins are prescribed in the setting of elevated lipid levels, it is plausible that the observed association may reflect underlying dyslipidemia rather than a direct effect of statin therapy. This highlights the importance of considering confounding variables, such as lipid levels, in evaluating the relationship between statin use and DSAP.

## Materials and methods

Using the TriNetX database, we conducted an updated retrospective cohort analysis (data run on 02/14/2025) to further evaluate this association. Patients with statin therapy exposure were identified using medication records, including atorvastatin, simvastatin, rosuvastatin, lovastatin, pravastatin, fluvastatin, and pitavastatin. A total of 970,166 patients receiving statin therapy were included in the initial cohort. 

To reduce confounding, propensity score matching was performed using a 1:1 nearest-neighbor approach, balancing cohorts on key demographic variables, including age, sex, race, and ethnicity. Balance diagnostics were assessed to ensure adequate matching before outcome analysis. Following matching, outcomes were evaluated, and ORs with 95% CIs were calculated using the Wald method. In the matched analysis, a total of 660 (0.068%) cases of DSAP (ICD-10-CM L56.5) were identified in the treated cohort, compared with 478 (0.049%) cases in the control cohort.

Comorbidities were assessed only after the initiation of statin therapy, ensuring that pre-existing conditions were excluded from the analysis. However, it remains uncertain whether all DSAP cases indeed developed after statin initiation, as retrospective electronic health record data may not capture pre-existing subclinical or unreported disease. Therefore, a limitation of this analysis is that TriNetX did not allow reliable confirmation of incident DSAP or minimum statin exposure duration before diagnosis. Furthermore, as DSAP is typically diagnosed by dermatologists, it remains uncertain whether all patients in the database underwent dermatologic evaluation. As a result, misclassification or miscoding of other porokeratosis variants as DSAP cannot be excluded. 

## Results

The association between statin use and DSAP (RR, 1.381; 95% CI, 1.227-1.553; p=0.0001) was statistically significant (Table 1). Nonetheless, this finding represents a correlation. Although the relative risk increase was statistically significant, the absolute risk difference between cohorts was small, with DSAP identified in 0.068% of statin users compared with 0.049% of controls, representing an absolute difference of 0.019%. Therefore, while the association may be clinically relevant in regards to understanding the dermatologic effects of statins, the overall likelihood of DSAP among statin users remains low. The observed association could be influenced by confounding factors such as underlying hyperlipidemia, sun exposure, immunosuppression, or socioeconomic status. Without accounting for these variables, the extent to which statin therapy itself contributes to DSAP risk remains uncertain. Further mechanistic studies are needed to reconcile these seemingly paradoxical findings.

**Table 1:** Demographic and clinical characteristics of statin users (cases) vs. non-users (controls) with associated risk of DSAP *After propensity score matching **Statistically significant values between cases and controls, defined at p<0.05 CI, confidence interval; DSAP, disseminated superficial actinic porokeratosis; RR, risk ratio; SD, standard deviation.

Characteristic	Cases (n=970,166)	Cases (%)	Controls (n=970,166)	Controls (%)	RR (95% CI)*	p-value*
Average age, SD (years)	61.5, 13.5		61.5, 13.8		-	0.475
Sex						
Male	414,218	42.70	398,433	41.10	-	0.92
Female	516,346	53.20	527,838	54.40	-	0.861
Race						
White	686,290	70.70	694,831	71.60	-	0.81
Black or African American	123,752	12.80	108,543	11.20	-	0.979
Asian	31,841	3.30	29,690	3.10	-	0.964
Native Hawaiian or Other Pacific Islander	7,633	0.80	9,846	1.00	-	0.636
American Indian or Alaska Native	2,902	0.30	3,517	0.40	-	0.812
Ethnicity						
Not Hispanic or Latino	689,086	71.00	675,025	69.60	-	0.746
Hispanic or Latino	78,677	8.10	78,885	8.10	-	0.585
Condition						
DSAP	660	0.07	478	0.05	1.381 (1.227-1.553)	<0.0001**

## Discussion

Several biologically plausible mechanisms may underlie the observed association between statin use and DSAP. Statins have been found to inhibit the mevalonate pathway, which plays a key role in keratinocyte differentiation and epidermal barrier function [5,6]. Beyond cholesterol synthesis, this pathway generates key intermediates such as isoprenoids, which are essential for protein prenylation, intracellular signaling, and regulation of keratinocyte proliferation and apoptosis [6]. It is plausible that disruption of these processes may impair normal epidermal turnover and promote the clonal expansion of abnormal keratinocytes, a proposed pathogenic mechanism of porokeratosis [4]. Interestingly, topical statin formulations have been reported as a potential porokeratosis treatment, supporting the concept that alterations in cholesterol supplementation may contribute to disease pathogenesis [4]. This raises the possibility that systemic inhibition of the mevalonate pathway by statins may paradoxically contribute to epidermal dysfunction in susceptible individuals, although causality remains unproven. This suggests that systemic and topical effects of statins may differ, possibly due to their delivery method, concentration, or interaction with cholesterol pathways in the skin.

This analysis is subject to several limitations. The reliance on RxNorm codes may have led to incomplete capture of comorbidities, potentially introducing bias into the data [1]. Furthermore, data could not be stratified by DSAP severity, affected body regions, or duration of statin therapy [3]. Additionally, environmental factors such as cumulative sun exposure were not controlled for. These are critical exposures in actinic dermatoses and could independently affect DSAP development [7]. Patients prescribed statins are more likely to be older and have increased healthcare utilization and cumulative environmental exposures, including ultraviolet radiation, which may disproportionately influence the observed association [8]. The observed association may also be influenced by differences in healthcare utilization between statin users and non-users. Patients receiving statins may have more frequent medical encounters, medication reviews, or specialist follow-up, increasing the likelihood that DSAP is recognized and coded. This potential detection bias may partially account for the observed association. Further research incorporating proxies for healthcare utilization, such as visit frequency or dermatology-focused encounters may help clarify the extend to which increased surveillance contributes to these findings. 

While we initially noted acne as a potential confounder, its relevance may lie in concurrent treatment regimens (e.g., isotretinoin, corticosteroids) or immune alterations that may overlap with pathways involved in DSAP. A cohort study that accounts for potential confounding variables-such as age, sex, race, prior or concurrent acne treatment, healthcare utilization, environmental and familial risk factors, and socioeconomic background-would have strengthened the robustness of our analysis [3].

## Conclusions

Despite these limitations, our findings provide insights into the safety profile of statins, particularly concerning their potential dermatologic effects. By highlighting a statistically significant correlation between statin use and DSAP, this study underscores the importance of continued vigilance in monitoring for adverse skin reactions. In practice, dermatologists may consider assessing statin use as part of the medication history in patients presenting with new or unexplained cases of DSAP.

However, while this association shows statistical significance, the absolute risk of DSAP among statin users remains low, and these findings do not establish causality. Further research is warranted to understand the underlying mechanisms and to guide clinicians in recognizing and managing these potential complications. Prospective studies with detailed clinical phenotyping, longitudinal follow-up, and stratification by lipid levels and cumulative sun exposure are needed to clarify causality. Furthermore, mechanistic investigations exploring cholesterol pathway modulation in keratinocytes may help assess whether this association reflects a pharmacologic effect of systemic statins or shared metabolic risk factors.
